# Industrial applications of fungal lipases: a review

**DOI:** 10.3389/fmicb.2023.1142536

**Published:** 2023-04-28

**Authors:** Ashish Kumar, Vinita Verma, Vimal Kumar Dubey, Alok Srivastava, Sanjay Kumar Garg, Vijay Pal Singh, Pankaj Kumar Arora

**Affiliations:** ^1^Department of Environmental Microbiology, Babasaheb Bhimrao Ambedkar University, Lucknow, India; ^2^College of Agriculture Sciences, Teerthanker Mahaveer University, Moradabad, Uttar Pradesh, India; ^3^Department of Plant Science, Faculty of Applied Sciences, MJP Rohilkhand University, Bareilly, India

**Keywords:** lipase, bioethanol, immobilization, biosensor, fungi

## Abstract

Fungal lipases (triacylglycerol acyl hydrolases EC 3.1.1.3) are significant industrial enzymes and have several applications in a number of industries and fields. Fungal lipases are found in several species of fungi and yeast. These enzymes are carboxylic acid esterases, categorized under the serine hydrolase family, and do not require any cofactor during the catalyzing of the reactions. It was also noticed that processes including the extraction and purification of lipases from fungi are comparatively easier and cheaper than other sources of lipases. In addition, fungal lipases have been classified into three chief classes, namely, GX, GGGX, and Y. Fungal lipases have applications not only in the hydrolysis of fats and oils (triglycerides) but are also involved in synthetic reactions such as esterification, acidolysis, alcoholysis, interesterification, and aminolysis. The production and activity of fungal lipases are highly affected by the carbon source, nitrogen source, temperature, pH, metal ions, surfactants, and moisture content. Therefore, fungal lipases have several industrial and biotechnological applications in many fields such as biodiesel production, ester synthesis, production of biodegradable biopolymers, formulations of cosmetics and personal care products, detergent manufacturing, degreasing of leather, pulp and paper production, textile industry, biosensor development, and drug formulations and as a diagnostic tool in the medical sector, biodegradation of esters, and bioremediation of wastewater. The immobilization of fungal lipases onto different carriers also helps in improving the catalytic activities and efficiencies of lipases by increasing thermal and ionic stability (in organic solvents, high pH, and temperature), being easy to recycle, and inducing the volume-specific loading of the enzyme onto the support, and thus, these features have proved to be appropriate for use as biocatalysts in different sectors.

## 1. Introduction

Triacylglycerol acyl hydrolases (EC 3.1.1.3) are also known as lipases and are recognized as a group of potential industrial enzymes, responsible for catalyzing the hydrolysis or breakdown of insoluble fats and oils (triglycerides), and they can release monoglycerides, diglycerides, glycerol, and free fatty acids over an oil–water interface (Geoffry and Achur, 2018; Patel and Shah, 2020). Moreover, lipases are carboxylic acid esterases that belong to the serine hydrolase family and do not require any cofactor to catalyze the reactions (Basheer et al., 2011). Lipases constitute the third biggest family of digestive enzymes after proteases and carbohydrates. They are a chief group of biocatalysts in the field of biotechnology (Demera et al., 2019; Lima et al., 2019). Furthermore, these enzymes in an organic medium are also able to catalyze synthetic (formation) reactions such as esterification, interesterification, alcoholysis, aminolysis, and acidolysis in addition to hydrolysis of triglycerides (Anobom et al., 2014; Lima et al., 2019). In both aqueous and non-aqueous mediums, lipases have high efficiency to catalyze reactions as they contain high stability against a high range of temperatures, pH, and even organic solvents. It is also known that lipases have a hydrophobic lid essential for their interfacial activity (Khan et al., 2017; Mehta et al., 2017; Tan et al., 2018; Bharathi and Rajalakshmi, 2019). Firstly, Clade Bernad, in 1856, had observed the lipase enzyme in pancreatic juice, where lipase performed the function of hydrolysis of oil and fats droplets and was capable of converting them into soluble digestible products (Jamilu et al., 2022). Lipases have been reported in animals, insects, and plants, as well as microorganisms such as bacteria, fungi, yeasts, and algae (Mehta et al., 2017; Sarmah et al., 2018; Bharathi and Rajalakshmi, 2019).

Lipases are highly diverse and mainly ubiquitous in animals, plants, and microorganisms (Bharathi and Rajalakshmi, 2019). Lipases that are specially derived from microbial sources have gained increasing attention in the industrial fields rather than those that are derived from animals and plants due to their suitable characteristic features and functional ability under highly difficult conditions and remain stable in organic solvents, chemical selectivity, enantio-selectivity, and do not need any cofactor to increase their catalytic efficiency during reactions (Bharathi and Rajalakshmi, 2019; Thapa et al., 2019).

Among microbial-origin sources of lipases, fungi have been recognized as good producers of extracellular lipases, and processes including extraction and purification are comparatively easier than other sources of lipases (Treichel et al., 2010; Geoffry and Achur, 2018). Because of their versatility, fungal lipases are recognized as Potential biocatalysts in both the industrial and biotechnological sectors. Fungal lipases have applications in several industries such as leather, textile, cosmetics, biodiesel production, detergent manufacturing, medicine and pharmaceutical, pulp and paper production, dairy product formation, beverages, medical and diagnostics, biosurfactant formation, fatty acid production, and the oleochemical industry (Kaur et al., 2016; Geoffry and Achur, 2018; Avhad and Marchetti, 2019; Jamilu et al., 2022).

The production of fungal lipases is largely affected by the composition of the medium, temperature, pH, inoculum volume, aeration, agitation, and several other factors. These other factors have been observed that affect the number of microbes, and several strategies have been applied that optimize the different parameters of the fermentation process by using statistical experimental designs (Kishan et al., 2013; Lima et al., 2019).

The review article mainly focuses on fungal lipases and their catalytic potential, sources of fungal lipases, classes of fungal lipases, different methods of fungal lipase immobilization, factors affecting the activity and production of fungal lipases, molecular techniques in fungal lipase production, and their industrial and biotechnological applications in various industries and sectors.

## 2. Bio-catalytic potential of fungal lipases

Fungi and yeast are distinguished as potential sources of fungal lipase among the different kinds of microorganisms (Tan et al., 2003). Since fungal lipases are substrate-specific and stable under a wide range of chemical and physical conditions, they have received significant attention in industries and other sectors, as fungi produce extracellular lipolytic enzymes that are easily extracted and purified, thus lowering production costs and making these preferred sources over bacterial lipases (Mehta et al., 2017).

Now days, the chief fungal strains, such as *Candida rugosa, Rhizopus oryzae, Mucor miehei, Rhizopus japonicus, Rhizopus arrhizus, Rhizopus delemar, Rhizopus niveus, Aspergillus niger*, and *Humicola lanuginosa*, are produced commercial lipases in their culture medium (Chandra et al., 2020). Fungal-originated commercial lipases have applications in several industrial sectors such as detergent formation, food and dairy products production, pharmaceutical and medicine, biodiesel production, oleochemical industries, bioremediation, the leather industry, bioremediation of wastewater, cosmetics and perfumeries, the medical and pharmaceutical sectors, ester synthesis, and the paper industry. Furthermore, some fungal lipases also have applications as diagnostic tools in the area of the medical field. Fungal lipases have tremendous potential as biocatalysts for biomolecule production due to their specificity and benefits for future development (Mehta et al., 2017; Geoffry and Achur, 2018; Mahfoudhi et al., 2022).

The major important advantages are as follows:

- They have high efficacy under mild reaction conditions.
- Easier to practice in the natural reaction medium and products.
- Capable to decrease contamination from the environment.
- Accessibility of lipases from diverse fungal sources.
- Enhancement of catalytic power of lipases through genetic engineering (Mahfoudhi et al., 2022).

## 3. Classes of fungal lipases

According to their protein topology, lipases were originally classified as bacterial lipases because of their high sequence diversity (Arpigny and Jaeger, 1999). There are eight families of lipases. The classification of lipase has been modified many times, and recently, there is a modified classification that contains 15 families, all of which are part of the ESTHER database available at http://bio0web.ensam.inra.fr/esther (Fu et al., 2013; Mahfoudhi et al., 2022). An ESTHER is an extensive database covering all the information on a huge variety of the α/β hydrolases fold superfamily that comprises lipases (Lenfant et al., 2012). An additional classification created on comparatively simplified lipase data has been recently published in the Lipase Engineering Database (LED), in addition to that by Arpigny and Jaeger (1999; http://www.led.uni-stuttgart.de). This classification now comprises not only bacterial lipases but also mammalian, fungal, and yeast lipases.

**Figure 1:**
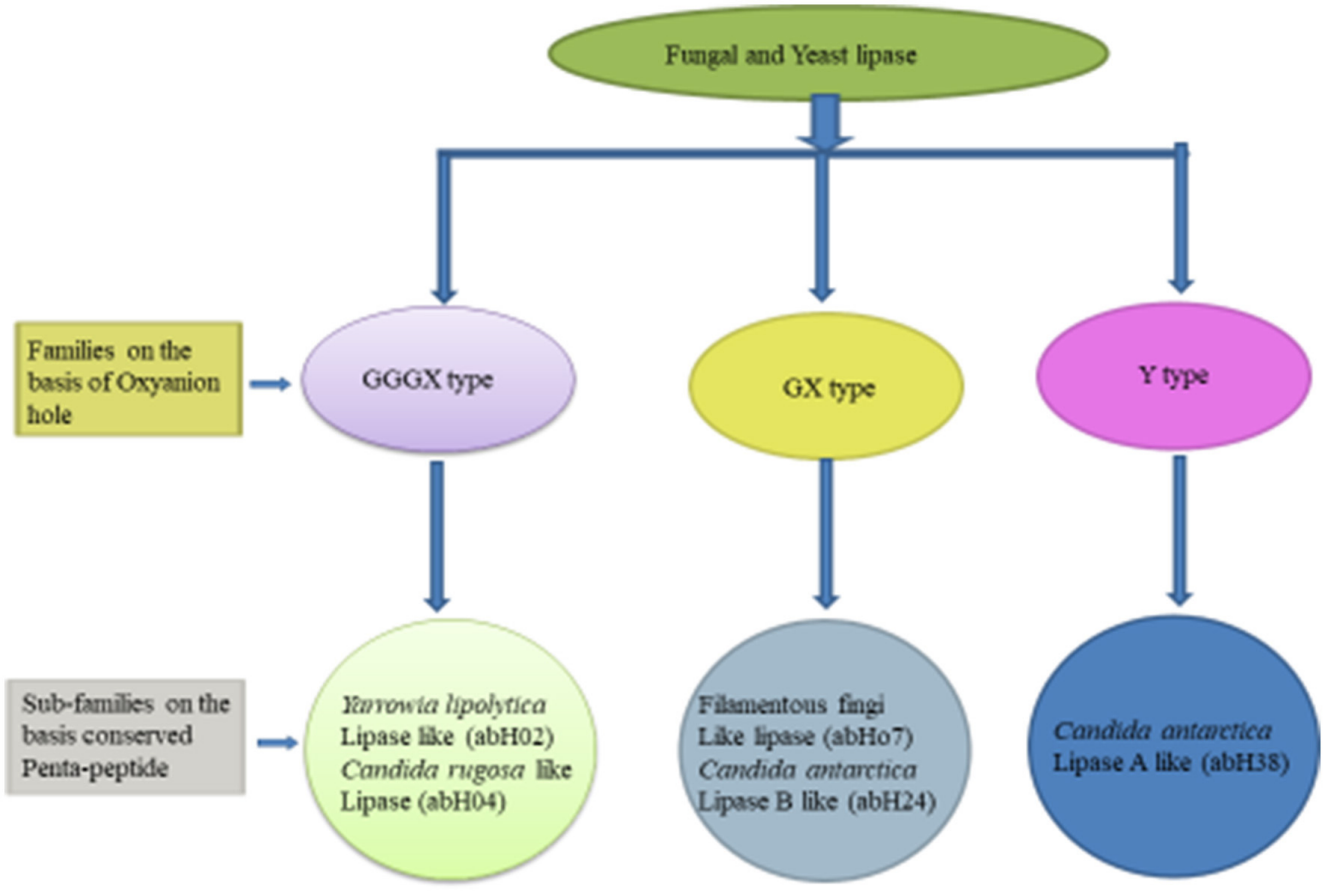
Classification of fungal and yeast lipase (Mehta et al., 2017; Mahfoudhi et al., 2022).

Based on the oxyanion hole pattern, this classification (shown in Figure 1) categorized these enzymes into three classes: GX, GGGX, and Y (Fischer and Pleiss, 2003). The backbone N–H of an amino acid (X) in a GX motif forms the first part of the oxyanion hole, whereas in GGGX types, the backbone N–H of the third glycine forms the first part, and later, a third type of oxyanion hole (Y-type) was discovered. A bulky amino acid, mainly tyrosine or aspartate, forms the oxyanion hole in Y-type hydrolases (Gupta et al., 2015). According to this classification and based on amino acid sequence similarity, yeasts and fungal lipases are classified into five subclasses. The GX class contains two subclasses, the GGGX class also contains two subclasses, and the Y class only contains one subclass (Borrelli and Trono, 2015; Gupta et al., 2015).

## 4. Sources of lipases

Among the various sources of lipases, the microbial sources of lipases have gained greater industrial attention because of their selectivity, stability, and broad substrate specificity (Kumar et al., 2016; Mehta et al., 2017; Mahfoudhi et al., 2022). Several microbes are recognized as potential sources of lipases, and these microbes are grouped as fungi, yeast, and bacteria. In this review, we discussed only the fungal and yeast sources of lipases.

### 4.1. Fungal sources of lipases

A large number of fungal strains are known to be capable of producing lipases that have distinctive catalytic properties that are crucial to various commercial applications (Pandey et al., 2016). In the commercial and industrial world, the most important fungal species that produce lipases are *Aspergillus* sp., *Penicillium* sp., *Rhizopus* sp., *Fusarium* sp., *Geotrichum* sp., *Trichoderma* sp., and *Mucor* sp. (Mohanasrinivasan et al., 2009; Bharathi and Rajalakshmi, 2019; Joshi et al., 2019). Moreover, some recently isolated fungi are also documented as they can produce lipase enzymes such as *Rhizopus oryzae* R1 (Helal et al., 2021), *Stemphylium lycopersici, Sordida* sp. (Rocha et al., 2020), *Aspergillus niger* 13 F, *Fusarium solani* 7 F (Patel and Shah, 2020), *Aspergillus flavus* (Ezema et al., 2022), *Aspergillus terreus* AH-F2 (Shabbir and Mukhtar, 2018), and *Thermomyces lanuginosus* (Tišma et al., 2019). The production of lipases by fungi is varied according to the fungal organism and composition of the growth medium and physical conditions such as the nitrogen source, carbon source, pH, and temperature (Pandey et al., 2016). Among the fungal sources, filamentous fungi are recognized as prominent lipase producers, and methods including extraction, purification, and processing are comparatively simple and easy when compared to other sources. Rocha et al. (2020) isolated two endophytic fungi, namely, *Stemphylium lycopersici* and *Sordida* sp., from the leaves of *Humiria balsamifera* and *Tocoyena bullata, and* both fungi have been observed to be capable of yielding the lipase enzyme of 397 U/ml/min and 286 U/ml/min, respectively. Shabbir and Mukhtar (2018) also isolated a fungus *Aspergillus terreus* AH-F2 from soil and oily samples that shows a maximum lipase production of 5.0 U/ml/min in a defined medium at pH 6.0. In another study, Patel and Shah (2020) isolated two fungi, namely, *Fusarium solani* 7 F and *Aspergillus niger* 13 F, and both the fungi are capable of producing 5.95 U/mL/min of crude lipase after 4 days of incubation at 30°C. Some fungal strains, their isolation sites, culture conditions, screening substrate, and specific enzyme activities are summarized in Table 1.

**Table 1:** The names of some fungi, their isolation sites, culture processes, and enzymatic activities.

**Fungal strain**	**Isolation site**	**Culture process**	**Specific activity**	**Opt. temp**.	**Opt. pH**	**References**
*Aspergillus sydowii* BTSS 1005	Soil sediment	Submerged fermentation	63.936 U/ml	NR	NR	Bindiya and Ramana, 2013
*Aspergillus niger*	Dairy effluent	Submerged fermentation	NR	NR	7.15	Colla et al., 2016
*Aspergillus niger* NCIM 584	NR	Solid-state fermentation	121.53 U/g des	30	6.87	Hosseinpour et al., 2012
*Aspergillus fumigatous*	Oil-contaminated soil sample	PDA plates with tributyrin and tween-80	2.35 U/ml	48	9.0	Mehta et al., 2018
*Talaromyces thermophiles*	NR	Mandels medium	9,868 ± 139 U mg^−1^	50	9.Lima	Romdhane et al., 2010
*Penicillium verrucosum*	Waste of babassu oil industry	Submerged fermentation	3.22 UmL^−1^	28	7.0	Pinheiro et al., 2008
*Fusarium incarnatum* KU377454	The locality of nearby Banasthali Vidyapith Rajasthan	Submerged fermentation	37.80 ± 0.55 IU mL^−1^	NR	NR	Joshi et al., 2019
*Rhizopus oryzae* R1	Soil samples from a gas station	NR	1.56 U/mg	40	6.0	Helal et al., 2021
*Antrodia cinnamomea* BCRC 35396	NR	Batch fermentation	26 U/ml	45	8.0	Shu et al., 2006
*Penicillium restrictum*	Babassu cake waste	Solid-state fermentation and submerged fermentation	17.4 U/g 12.1 U/g	NR	NR	de Azeredo et al., 2007
*Fussarium solani* 7 F	Cotton seed soapstock	Submerged fermentation	5.95 U/mL/min	30	7.0	Patel and Shah, 2020
*Aspergillus niger* MTCC 872	NR	Solid-state fermentation	28.19 u/gds 28.19 u/gds	40	6.0	Nema et al., 2019
*Trichoderma* sp.	Biopharmaceutical oil waste	Solid-state fermentation	93.07 U	NR	NR	Mohanasrinivasan et al., 2009
*Penicillium* sp.	Biopharmaceutical oil waste	Solid-state fermentation	199.696 U	NR	NR	Mohanasrinivasan et al., 2009
*Aspergillus niger C*	Soil sample	Selective medium with olive oil	7.3 U/mL	NR	NR	Kashmiri et al., 2006
*Aspergillus niger C*	NR	Submerged fermentation	13.12 U mL^−1^	55	5.5	Lima et al., 2019
*Aspergillus niger* 13 F	Cotton seed soapstock	Submerged fermentation	4.2 U/mL/min	30	7.0	Patel and Shah, 2020
*Aspergillus terreus* AH-F2	Soil and oily sample	Submerged fermentation	5.0 U/mL/min	30	6.0	Shabbir and Mukhtar, 2018
*Penicillium Simplicissimum*	NR	Solid-state fermentation	19.6 U/mg	50	5.0	Gutarra et al., 2009
*Aspergillus flavus*	Decaying seed of Cucameropsis mannii	Phenol red containing agar plates	NR	NR	NR	Ezema et al., 2022
*Rhizopus* sp.	Coconut pulp	Solid-state fermentation	75 IU/g IS	NR	NR	Martínez-Ruiz et al., 2008
*Aspergillus awamori* BTMFW 032	Seawater	Submerged fermentation	1,164.63 U/mg protein	40	7.0	Basheer et al., 2011
*Rhizopus homothallicus* IRD 13a	NR	Solid-state fermentation	10.700 U/mg	40	7.5	Diaz et al., 2006
*Rhizopus oryzae*	NR	*Rhizopus oryzae*	3.919 U/g	NR	NR	López et al., 2010
*Fussarium solani* strainSKWF7	Dairy wastewater	Glucose-yeast extract peptone broth with olive oil	73.3 U/ml	NR	NR	Kanmani et al., 2013
*Penicillium roqueforti* ATCC 10110	NR	Solid-state fermentation	14.67 ± 1.47 U g-1	NR	NR	Araujo et al., 2022
*Penicillium chrysogenum*	Soil sample	Basal GKM medium	68 Units mL^−1^	30	5.0	Bancerz et al., 2005
*Penicillium verrucosum*	Waste of babassu oil industry	Solid-state fermentation	40 U/g of dry bran	44	7.0	Kempka et al., 2008
*Aspergillus* sp.	Biopharmaceutical oil waste	Solid-state fermentation	213.165 U	NR	NR	Mohanasrinivasan et al., 2009
*Rhizopus sp*.	Soil sample	PDA plate	28.8 U/mL	35	7.0	Al-shawawreh, 2021
*Penicillium aurantiogriseum*	Soil sample	Tributyrin agar plate	16.718 IUml^−1^ min^−1^	30	6.8	Pandey et al., 2016
*Penicillium sp*.	Oil mill effluent	Potato dextrose agar	3.911 IU mL^−1^	NR	NR	Rihani et al., 2018
*Penicillium chrysogenum*	NR	Solid-state fermentation	650 U/gds	40	9.0	Rajeswari et al., 2011
*Thermomyces lanoginosus*	NR	Solid-state fermentation	391 U gdb^−1^	40	9.0	Šibalić et al., 2020
*Rhizopus oryzae*	NR	Solid-state fermentation Submerged fermentation	50 U/ml 62.67 U/ml	NR	NR	Hermansyah et al., 2019
Aspergillus aculeatus	Dairy waste contaminated soil	Potato Dextrose agar	9.51 U/ml	NR	NR	Roy et al., 2021
*Aspergillus aculeatus*	Non-dairy creamer industrial waste	Modified phenol red agar plate	0.44 ± 0,03 U/mg biomass	30	7.0	Triyaswati and Ilmi, 2020

### 4.2. Yeast sources of lipases

Yeast-derived lipases have distinctive applications in the chemical and pharmaceutical industries and in biodiesel production (Singh and Mukhopadhyay, 2012). Recently, the reviewed literature survey demonstrated that *Yarrowia* sp., *Cryptococcus* sp., *Candida* 99–125, *Meyerozyma guilliermondii, Candida utilis, Magnusiomyces capitatus* JF5, *Pichia* sp. *Rhodotorula* sp., *Candida rugosa*, and *Candida utilis* are very good primary producers of lipases (Dalmau et al., 2000; Dominguez et al., 2003; Tan et al., 2003; Thirunavukarasu et al., 2016; Bharathi and Rajalakshmi, 2019; Knob et al., 2020; Salgado et al., 2020). According to the literature review, *Candida* sp. is the lipase-producing category of yeasts with the most potential. The lipase from *Candida* sp. has been widely recognized for its structural, biochemical, and catalytic properties.

Thirunavukarasu et al. (2016) reported that *Cryptococcus* sp. can produce a maximum lipase activity of 753 ± 19 U/g dry substrate (gds) after growing on agro-industrial residues. Recently, Knob et al. (2020) observed that after culture conditions using statistical design, *Meyerozyma guilliermondii* showed lipase activity of 285.82 U ml^−1^ when grown in a 2% of cheese whey at 4.0 pH for 24 h. Some names of yeasts, isolation sites, culture processes, and enzymatic activities are summarized in Table 2.

**Table 2:** Some names of yeasts, their isolation sites, culture processes, and enzymatic activities.

**Yeasts**	**Isolation site**	**Culture process**	**Specific activity**	**Opt. Temp**.	**Opt. pH**	**References**
*Yarrowia lipolytica* CECT 1240	NR	Solid-state culture	23 kUI^−1^	NR	NR	Dominguez et al., 2003
*Crytococcus* sp MTCC 5453	NR	Solid-state fermentation	753 ± 19 U gds^−1^	NR	NR	Thirunavukarasu et al., 2016
*Meyerozyma guilliermondii*	Slaughter house fridge and oil mill effluent	Submerged fermentation	285.82 U mL^−1^	40	3.0	Knob et al., 2020
*Candida rugose*	NR	Batch culture	4.3 U/mg biomass	NR	NR	Dalmau et al., 2000
*Candida* 99–125	Soil sample of fish processing industry	Wort agar slant	8,060 U mL^−1^	28	7.0	Tan et al., 2003
*Magnusiomyces capitatus* JT 5	Olive mill wastewater	Yeast malt agar	1.4 U/mL	NR	NR	Salgado et al., 2020
*Trichosporon asahii* MSR 54	Petroleum sludge sample	Selected M2 medium	104 U/ml	40	9.0	Kumar and Gupta, 2008
*Guehomyces pullulans*	NR	Liquid culture medium	0.27 U/mL	40	8.0	Demera et al., 2019
*Thermomyces lanuginosus*	NR	Solid-state fermentation	NR	40	NR	Tišma et al., 2019
*Candida W 3.8*	Wonorejo mangrove area	Tween-80 agar medium	2,357.3 U/mL	45	7.0	Alami et al., 2017

NR= Not Reported.

## 5. Immobilization of fungal lipase

The term immobilization of enzyme designates an enzyme that is either physically or chemically confined or localized in a certain space or region with a plenteous holding of its catalytic activity in several cycles to follow (Mohamad et al., 2015). The immobilization of enzymes can enhance enzyme stability and catalytic power (Liese and Hilterhaus, 2013; Thangaraj and Solomon, 2019). There are several methods or techniques for the immobilization of enzymes by using different supports, and these techniques are either reversible (physical adsorption) or irreversible (membrane confinement or encapsulation, cross-linking, entrapment, and covalent-binding; Bilal et al., 2021).

Physical adsorption is an old, simple, and less costly technique involved in the binding of atoms, ions, or molecules of a dissolved solid, liquid, or gas to the surface region of a carrier by weak interactions such as Landon–van der Waals interactions, hydrogen bonding, ionic bonding, and hydrophobic interactions (Ahmad and Sardar, 2015). In contrast, covalent bonding is an irreversible method that involves the binding of the enzyme to a carrier through strong interactions during catalytic reactions and improves the stability of the enzyme automatically (Zheng et al., 2012), while the membrane-confining or encapsulation technique entraps the enzyme within a semi-permeable spherical membrane with a selectively controlled permeability (Nedovic et al., 2011; Thangaraj and Solomon, 2019). In the entrapment technique, the enzyme is enclosed within the lattice of a polymer matrix or membrane without a chemical reaction. Furthermore, the cross-linking process involves the formation of physical or chemical intermolecular cross-linkages between a protein and other protein molecules or a functional group of an insoluble matrix to form a three-dimensional structure. These techniques have made great advancements in fungal lipases, such as thermal or ionic stability, easy recycling, and volume-specific loading of the enzyme onto the support, and thus immobilization can improve the catalytic activity of lipases to apply them in different areas.

Asmat et al. (2019) immobilized the lipase of *Candida rugosa* on multi-walled carbon nanotubes (MWCNTs) as a carrier material by covalent bonding through glutaraldehyde, and the immobilized lipase showed high catalytic activity of about 3-fold of free lipase, exhibited high thermal stability in a broad range of pH, and was used to synthesize isoamyl and ethyl butyrate, which have characteristic banana and pineapple flavors, respectively. Similarly, Bayramoglu et al. (2022) also immobilized the *Candida rugosa* lipase by using magnetic chitosan beads as a carrier by Schiff base reaction, and the immobilized lipase enzyme showed better thermal and storage stability at various temperatures than free lipase and was capable of synthesizing isopentyl acetate and isoamyl acetate from isopentyl alcohol and isoamyl alcohol through the esterification reaction in the hexane medium. In another similar study, Aghaei et al. (2021) also immobilized the *Candida rugosa* lipase by cross-bonding on epoxy-activated Cloisite 30 B as a carrier and successively used it in the olive oil hydrolysis and synthesis of the isoamyl acetate (banana flavor) and biodiesel production at ~95.4%. Moreover, Gricajeva et al. (2018) immobilized the lipase (Resinase A 2x) of *Aspergillus* sp. by using cross-linking on the magnetic particles as a support and found that it has applications in the synthesis of 2-phenylethyl butanoate (fragrance compound and flavor). Lira et al. (2021) also immobilized the lipase of *Thermomyces lanuginosus* on agro-industrial wastes as a support and found that it has applications in the production of hexyl laurate.

## 6. Factors that affect the production and activity of fungal lipases

Several factors strongly affect the production and catalytic activity of lipases, and the presence of several inducers, such as carbon sources, nitrogen sources, pH, and temperature, are the major factors considered.

### 6.1. Carbon source

The production of lipases from fungi largely depends on the induction of lipase enzyme-encoding genes in microbes. Therefore, carbon sources play an important function in the stimulation of lipase enzyme-encoding genes in all kinds of microbial lipase-producing sources (microorganisms). Carbon sources help in the improvement of the fermentation process, which, in turn, increases cellular metabolism and results in increased fungal lipase activity (Alabdalall et al., 2020).

However, several oily carbon sources, such as olive oil, palm oil, and other vegetable oils, have been used as inducers for lipase enzyme production in microbes. Sethi et al. (2013) observed that when mustard seed oil was used as a carbon source in the culture medium of *Aspergillus terreus*, it showed a significant yield of lipase. Fatima et al. (2021) also observed that when an amalgam of olive oil cake and sugar cane bagasse is used as a carbon source, an increased production of lipase is attained by fungal strains. Olive oil cake has been found to be a good inducer of the lipase enzyme when compared to other carbon sources (Fatima et al., 2021). Different lipid sources, such as mustard oil, neem oil, coconut oil, olive oil, sunflower oil, palm oil, and cucumber oil, and many non-lipid sources of carbon such as sucrose, lactose, maltose, glucose, galactose, xylose, fructose, and mannitol, have been used in the media of *Aspergillus sydowii* to check the effect of these sources for lipase production by Bindiya and Ramana (2013). Carbohydrates act as monovalent carbon sources for lipase production, due to which, in the presence of starch and sucrose, a low yield of lipase was achieved by *Aspergillus niger*, but when fructose was used as a carbon source, it showed high lipase production of 777.44 U/ml (Alabdalall et al., 2020). Tween 80 has been used to assist in improving the recovery of lipase in *Acinetobacter* sp. (Li et al., 2001; Mahfoudhi et al., 2022).

### 6.2. Nitrogen source

In the synthesis of lipase enzymes, nitrogen sources also have an important role because these are necessary for the growth of microbes and the stimulation of lipase production in microbes. Different organic and inorganic nitrogen sources, such as peptone, tryptone, sodium nitrate, ammonium salts, urea, and yeast extract, have a major role in the production of lipase in various microorganisms (Oliveira et al., 2016; Bharathi and Rajalakshmi, 2019; Priyanka et al., 2019; Fatima et al., 2021). Higher lipolytic activity was demonstrated by *Rhizopus* sp. showed higher lipolytic activity, when added urea in their growing medium (Rodriguez et al., 2006; Mehta et al., 2017). Similarly, an amalgam of peptone and another nitrogen extract has been utilized for the production of lipase from *Aspergillus* sp. (Bharathi and Rajalakshmi, 2019; Colonia et al., 2019). It was also observed that a mixture of nitrogen sources influences lipase enzyme activity. *Trichoderma harzianum* achieved maximum lipase activity when glucose and peptone acted as sources of carbon and nitrogen in their culture medium, whereas minimum lipase activity was observed with glucose and yeast extracts as sources of carbon and nitrogen, respectively. Furthermore, Bindiya and Ramana (2013) used different nitrogen sources with a concentration of 1% w/v for investigating their effects on lipase production in *Aspergillus sydowii*. The nitrogen sources used in the study were (NH_4_)_2_SO_4_, yeast extract, NaNO_3_, tryptone, KNO_3_, NH_2_CONH_2_, beef extract, and NH_4_Cl. The highest activity of lipase output was achieved when using NH_4_Cl.

### 6.3. Temperature

Temperature also has a significant effect on microbial lipase production and can change the physical properties of cell membranes, resulting in the influencing of the secretion of the extracellular enzyme. An optimum temperature is crucial and has a vital role in the secretion of enzymes in the shake-flask method. A higher biomass output of lipase production was achieved at a temperature of 37°C (Mehta et al., 2017; Bharathi and Rajalakshmi, 2019). Researchers recorded that a small increase in temperature at 38°C stimulates the output of lipase (Bharathi and Rajalakshmi, 2019; de Souza et al., 2019). It has been observed that a lower temperature decreases the output of the lipase enzyme, while a higher temperature also affects its activity. In a research study, Mukhtar et al. (2016) studied the effect of different incubation temperatures ranging from 25 to 55°C on the production of lipase enzyme from *Aspergillus niger*. The highest output of lipase was obtained at 30°C followed by 35, 40, 25, 50, and 55°C. In another study, Mahmoud et al. (2015) investigated the effect of various incubating temperatures, viz., 10, 20, 30, and 45°C, on the production of lipase from *Aspergillus terreus*. The maximum activity of lipase was achieved at 45°C (15 U/mL), which was decreased with the decrease in temperatures, viz., 30°C (12 U/ml), 20°C (9.5 U/ml), and 10°C (3.0 U/ml).

Comménil et al. (1995) mentioned that the *Botrytis cinerea* strain produced a temperature-sensitive fungal lipase that achieved the highest activity at 38°C and was completely inactivated above 60°C. Furthermore, the *Botrytis cinerea* lipase can also be stable at room temperature, with 98% of its initial activity observed after an incubation period of 48 h. *Nomuraea rileyi* and *Rhizopus oryzae* showed their highest enzymatic activities at 60°C, and great stabilities were noted at 50°C (Supakdamrongkul et al., 2010; Saranya and Ramachandra, 2020). Recently, a lipase was isolated and purified from *Cladosporium tenuissimum*, and the lipase achieved its maximum activity at 60°C (Saranya and Ramachandra, 2020).

### 6.4. The pH of the medium

In general, bacterial-origin lipases have either an alkaline pH or neutral pH. Recent research reported that conditions with an alkaline pH or a slightly neutral pH stimulated the production of lipase in both bacteria and fungi (Ramakrishnan et al., 2016; Bharathi and Rajalakshmi, 2019). According to Taskin et al. (2016), *Rhodotorula glutinis* HL 25 was observed with fair lipase activity when the pH of their lipase-producing medium was kept slightly neutral. On the other hand, acidic pH stimulates the production of fungal lipases. Turati et al. (2019) recorded the enhanced output and activity of fungal lipase when the pH of the reaction process was kept at 4.0. Mahmoud et al. (2015) investigated the effect of different pH ranges from 2.0 to 12 on the production of lipase from *Aspergillus terreus*; it showed maximum enzyme activity at pH 8.0.

### 6.5. Surfactants

Surfactants are ionic, long-chain organic molecules with a hydrophobic part and a hydrophilic part, and they can increase the permeability of the cell membrane, thus facilitating the export of many other molecules across the membrane and also stimulating the secretion of the protein. Surfactants are capable of changing the internal charges of lipase from cationic to anionic and vice versa. Due to this, the physicochemical properties of lipases are changed (Essamri et al., 1998). Surfactants are important gradients for emulsion preparations during the lipase assay at each step of enzyme production, purification, and their characterization (Supakdamrongkul et al., 2010). According to Saranya and Ramachandra (2020), several lipases revealed deviation in their affinities to the substrate when surfactants, such as Triton X-100, Tween 20, SDS, and Tween 80, were used in different concentrations. Lipase produced by the fungus *Nomuraea rileyi* showed enhanced activity in the presence of Tween 80 and SDS, whereas *Rhizopus oryzae*, a thermophilic fungus, showed less activity when Tween 80 and SDS were added (Supakdamrongkul et al., 2010).

In general, surfactants, such as Tween 80, Tween 20, Triton X-100, and cetyltrimethylammonium bromide (CTAB), enhance the activity of lipases when they are incorporated into the fermentation medium, but their enhancing effects may be varied from surfactants to strains (Silva et al., 2005; Niaz et al., 2013; Das et al., 2017; Geoffry and Achur, 2018). Thus, the selection of an appropriate surfactant must be important for obtaining high lipase activity.

### 6.6. Moisture content

Moisture content plays a major role in fungus growth and the production of lipases, especially in solid-state fermentation processes (Singhania et al., 2009). Moisture affects the physical properties of the solid substrate for several reasons, such as decreasing substrate porosity, altering the particle structure of the substrate, promoting the development of stickiness, reducing gas volume and aeration, and limiting the diffusion of oxygen in the substrate layer (Mukhtar et al., 2016; Oliveira et al., 2016).

High moisture content ultimately decreases the filamentous growth of fungi and thus results in a decrease in the activity and production of lipases. Optimum moisture content enhances lipase production and hydrolytic activity.

### 6.7. Metal ions

Metal ions are a significant ingredient and play different roles in influencing the structure and working of enzymes including lipases (Mahfoudhi et al., 2022). Different fungal lipases showed different behaviors toward different metal ions. Lipase produced by *Aspergillus japonicas* is inhibited when incubated with Mn^2+^ and Hg^2+^, whereas it remains stable when incubated with Ca^2+^ (Jayaprakash and Ebenezer, 2012). Similarly, Katiyar and Ali (2013) noticed that increased activity of lipase has been shown by *Candida rugosa* in the presence of Ca^2+^ ions. Interestingly, Saranya and Ramachandra (2020) reported that there is no effect of Ca^2+^, Mg^2+^, Na^+^, and K^+^ on the activity of lipase produced from *Cladosporium tenuissimum*. Toida et al. (1995) also noticed that metal ions, such as Cu^2+^, Fe^3+^, Hg^2+^, Zn^2+^, and Ag^+^, inhibited the lipase activity isolated from *Aspergillus oryzae*.

## 7. Molecular techniques in lipase production

The increased industrial application of lipases is responsible for the scale-up in the productivity of lipases, and several approaches have been practiced to fulfill this aim. In this section, we resume several processes and metabolic engineering techniques to carry out fungal lipase production. Industrial lipases are chiefly produced from fungi extracellularly. There have been several studies conducted on the production of free-type lipases, focusing primarily on the parameters related to optimizing the medium and operating conditions in batch cultures. Fed-batch fermentation has recently resulted in a significant increase in lipase productivity. Low productivity and high cost are generally associated with wild-type lipases. They naturally lose the optimal specificities and required properties for industrial feedstock. Hence, to obtain the standards of quantity and manipulation of industrial processes, we try to opt for other substitute methods, such as cloning and expression of the recombinant genes of lipases, to find an appropriate quantity of pure biocatalysts (Mahfoudhi et al., 2022).

Codon optimization in the lip3 gene of *Candida rugosa* can improve lipase production by ~50- to 70-fold (Chang et al., 2006). In a study, Hwang et al. (2014) indicated that promoter optimization is a generally used technique, which significantly advances the production of lipases. Techniques such as gene modification can make genes adjustable for expression within the recombinant host cells. Additionally, Yaver et al. (2000) have engendered insertion mutant libraries in a recombinant *Aspergillus oryzae* strain capable of expressing the *Thermomyces lanuginosus* lipase using restriction enzyme-mediated integration as a mutagen, and the results have proved that genetic modification can be used to modulate the expression of heterologous proteins. It is possible to improve lipase production using recombinant lipase, but its yield is limited by many post-translational events, such as disulfide bond formation, solubility, miss-folding, secretion, proteolysis, and even toxicity to host cells (Makrides, 1996; Mahfoudhi et al., 2022). To overcome these limitations, genetic and metabolic engineering can play an important role in increasing the production of recombinant lipase. Scaling up fermentation through efficient and convenient techniques can enhance lipase production. By optimizing fermentation conditions, Zhao et al. (2008) significantly enhanced the production of the *Candida rugosa* lipase in constitutive recombinant *Pichia pastoris* in both laboratory and pilot scales. In this experiment, a pH-stat and two-stage fermentation strategy combined with exponential feeding was used to scale up fermentation from 5 to 800 L, which achieved an excellent balance between the expression of recombinant lipase and the growth of the host cells. In the 800 L scale, an optimal lipase activity of ~14,000 IU/ml and a cell wet weight of 500 g/L have been determined. It is possible to effectively control the cell growth rate in large-scale fermentations of recombinant lipases by tuning their cell lyse and proteolytic sensitivity of lipase (Narayanan and Chou, 2009). Significant interest has been shown in improving biofuel production by upgrading lipase production through metabolic engineering (Atsumi and Liao, 2008; Lee et al., 2008). Zhou et al. (2019) cloned the *Rhizomucor miehei* lipase gene (rml) in *Yarrowia lipolytica* Polg and obtained 19.5 U/ml lipase activity; after codon optimization of the rml gene, an increased lipase activity (26.9 U/ml) was obtained. Subsequently, a method was developed for constructing hybrid promoters harboring different copy numbers of the upstream activation sequences fragment (UAS1B), and the recombinant strain Po1g/hp12d-*rml* 25# reached 38.9 U/ml. Wang et al. (2018) increased the 48.7% recombinant lipase activity of *Galactomyces geotrichum* in *Pichia pastoris* through codon optimization of the lipase gene.

## 8. Industrial and biotechnological applications of fungal lipases

**Figure 2:**
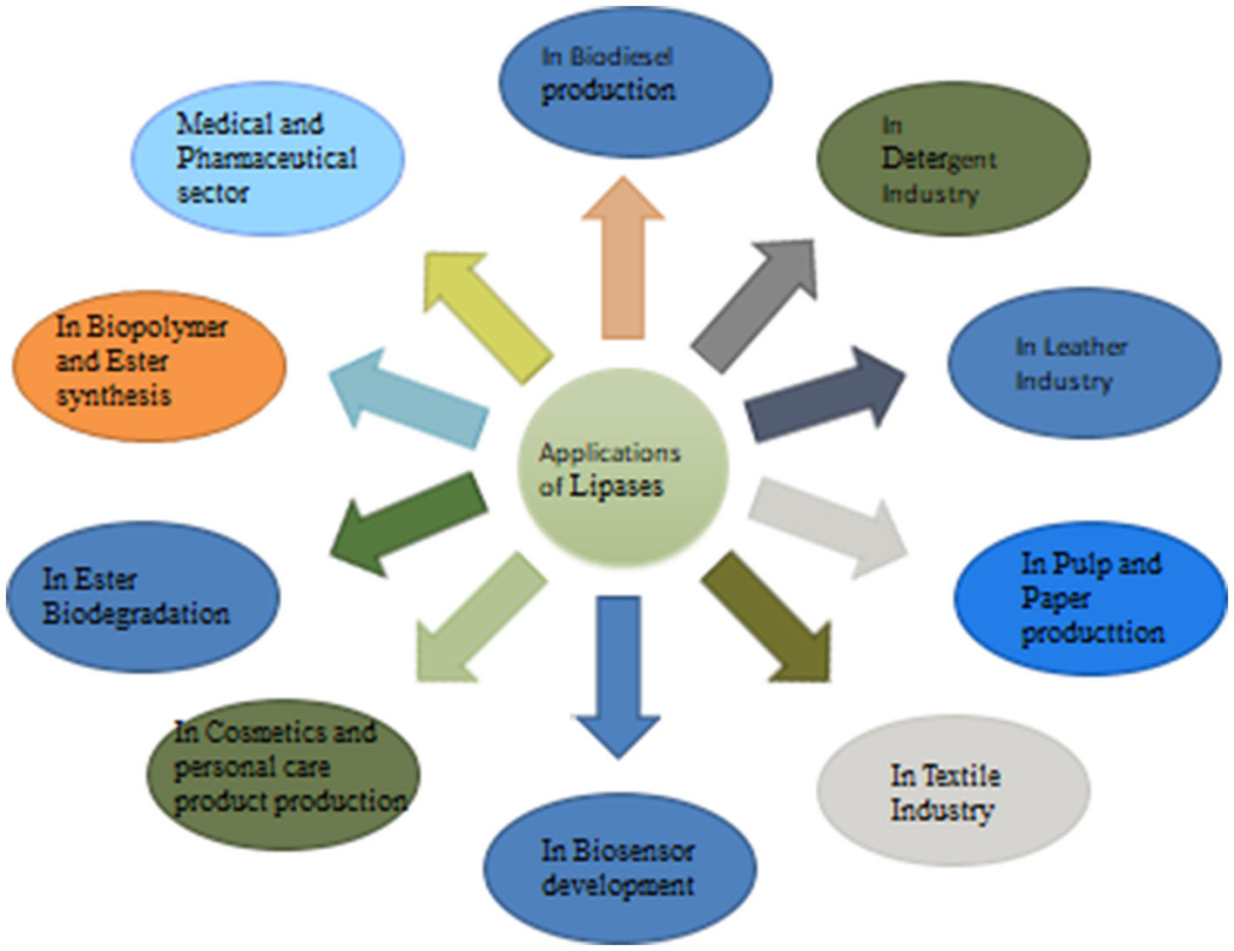
Applications of fungal lipases.

Fungal lipases are widely known for their enzymatic properties and substrate specificity, which make them versatile tools for industrial applications. They have become a chief group of biotechnological enzymes because of the versatility of their properties and easy mass production. Figure 2 represents the systematic applications of fungal lipases.

### 8.1. In biodiesel production

Continuous steady growth in population, transportation, and industrialization in the world results in the origin of increased demand for energy for industrial and domestic uses (Santos et al., 2020). To fulfill this increased demand for energy, we depend mainly on fossil fuels (petroleum-derived diesel), but the reservoir of fossil fuels (petroleum-derived diesel) is limited in quantity and will be completely exhausted in the future. A high amount of CO_2_, CO, and NOx gases is released into the environment by the burning of diesel, which in turn increases pollution and produces a greenhouse gas effect on the Earth (Quayson et al., 2020; Almeida et al., 2021). Therefore, we should try to search for alternative renewable sources of energy that could fulfill our needs for energy in the upcoming future. Biodiesel could be an effective alternative resource for our energy needs and has many advantages over fossil fuels (Santos et al., 2020). Biodiesel is a mixture of fatty acid alkyl esters and is non-toxic, biodegradable, and has low carbon, sulfur, and high hexadecane contents. Therefore, biodiesel produces low carbon dioxide (CO_2_), carbon monoxide (CO), and nitrogen oxide (NOx) emissions in comparison to petroleum-derived diesel (Almeida et al., 2021). Moreover, in this way, we can reduce pollution and greenhouse effects by using increased amounts of biodiesel. Many methods of biodiesel manufacturing, such as dilution, pyrolysis, micro-emulsion, and transesterification, are used. The substrates, such as animal fats (bovine tallow and lard), greases (trap grease and float grease), used cooking oils, and vegetable oils (rapeseed, cotton seed, palm, soybean, corn, jatropha, peanut, sunflower, and canola), are used for biodiesel production by transesterification. Transesterification can be either catalytic or non-catalytic, and hence, the production of biodiesel by using enzymes has become popular now due to its low energy consumption and mild reaction conditions (Vilar et al., 2018; Pérez et al., 2019; Carvalho et al., 2020). Transesterification reaction requires a small chain alcohol (methanol, ethanol, and propanol), a catalyst (acid, base, and catalyst), and either vegetable oil or animal fats (Almeida et al., 2021).

Enzymes, especially lipases, are used as a catalyst for biodiesel production in the transesterification process. Lipases have high catalytic activity, regioselectivity, and stereoselectivity in mild physical conditions such as pH, temperature, and pressure.

Several yeast and fungal lipases have catalyzed the production of biodiesel, including *Aspergillus niger, Rhizomucor miehei, Ralstonia* sp, *Candida rugosa, Candida antarctica, Rhizopus oryzae, Thermomyces lanuginosa*, and *Magnusiomyces capitatus* (Fan et al., 2012). Winayanuwattikun et al. (2011) immobilized the lipase of *Candida rugosa* B on seabeds EC-OD and used it as a biocatalyst for the synthesis of enzyme-catalyzed biodiesel. Similarly, Tian et al. (2021) reported that *Rhizomucor miehei* lipase (RML), with only a single chain of α/β type protein, is largely used in the preparation of biodiesel, due to its high catalytic activity and tolerance against methanol.

### 8.2. In detergent manufacturing

A detergent is either a sodium salt of long-chain benzene sulfonic acid or a sodium salt of long-chain alkyl hydrogen sulfate that possesses cleansing properties in water, as is the case with soaps. Both anionic groups (sulfate or sulfonic) and non-ionic groups (long-chain hydrocarbon) are present in a detergent; these groups make a detergent more soluble in water. Detergent is a water-soluble cleansing agent that combines dirt and impurities and makes them more soluble in water without forming scum with the salt of hard water. The use of detergents in washing is increasing the popularity of laundry and household detergents, because they are anti-static, dispersible in water, and give softness to fabric. Several brands of laundry and household detergents with different properties are now available in the market (Verma et al., 2012). Higher use of detergents increases the detergent load in the environment and becomes a pollutant. Moreover, these detergents also have a higher washing temperature. The use of hydrolytic fungal lipases as additives in the preparation of household and laundry detergents is commercially important, because lipases can decrease the detergent product load from the environment by saving energy by lowering the washing temperature of the detergent that is to be used (Mehta et al., 2017). Navo Nordisk, in 1994, first introduced the first commercial lipase. Lipolase was produced by the fungus *Thermomyces lanuginosus* and has been expressed in *Aspergillus oryzae. Thermomyces* sp. produces the most significant detergent by using the lipases that are primarily used in Lipolase and Novozymes (Gillis, 1988; Mehta et al., 2017). Furthermore, these lipases, including *Humicola lanuginosa, Talaromyces thermophilus, Rhizopus oryzae*, and *candida* sp., are also used in the preparation of detergents that promise better washing performance and energy savings (Nishioka et al., 1990; Salleh et al., 1993; Jaeger et al., 1994; Romdhane et al., 2010; Mahfoudhi et al., 2022). Wang et al. (2018) reported an alkaline, low-temperature, and strong surfactant tolerance recombinant lipase of *Trichoderma lentiform* ACCC30425 in *Pichia pastoris* GS115; this lipase is suitable for use in the detergent industry. Xiao et al. (2021) also reported a recombinant lipase Lip486 without clear homology alao to know lipase, Lip486 was discovered by using functional metagenomics technology and belongs to a new sub-familycalled lipolytic enzyme family II. The recombinant Lip486 expresses great activity and stability even in alkaline pH and medium–low temperature environments; these properties will make the lipase most important for use in detergent and other industries in the future.

### 8.3. In the leather industry

Leather industries are involved in the manufacturing of goods, such as bags, shoes, footwear, garments, belts, and purses, and have a considerable role in the world's economy and foreign exchange earnings and employment (Dixit et al., 2015; Khambhaty, 2020). However, these industries also harm the environment by releasing several toxic chemicals that are used during various steps of the tanning process. Leather industries have been established in a large number over the past few years, because leather and leather products have significant importance in the world's fashion industry. Leather production involves the conversion of raw animal skin and hides into leather by a series of different mechanical and chemical processes, such as soaking, dehairing, bating, degreasing, and post-tanning. Various chemicals are used in different processes of tanning that are released as waste, contributing to the significant increase in chemical oxygen demand (COD), total dissolved solids (TDSs), sulfates, chlorides, and heavy metals to the wastewater to be released into water bodies such as rivers, lakes, and the sea, thus polluting them enormously (Dayanandan et al., 2013; Dixit et al., 2015; Khambhaty, 2020). In order to reduce the application of these toxic chemicals, enzymes have been recognized as the best practical alternative to use during tanning, and these enzymes can help in waste management (Khambhaty, 2020). Lipases are an important group of enzymes in the various processes of leather production, because animal skin and hides contain protein and fat in collagen fibers. These skin and hides must be partially and totally removed before they are tanned. Lipases especially break down the lipids without damaging the leather and show the degreasing process with lower environmental impact (Choudhary et al., 2004; Dayanandan et al., 2013; Mehta et al., 2017).

Moreover, fungal-origin lipases are more efficient when compared to those originating from animals and plants as they are characterized by high yield, the rapid growth of fungi on cheaper media, and more stable, easy, and safer production methods. In a study, *Aspergillus tamarii* MTCC5152 was recognized for its high-level production of lipase (758 ± 3.61 u/g) through solid-state fermentation, the crude lipase (3%) used for tanning fleshing, and its 92% fat solubility (Dayanandan et al., 2013). Similarly, in another study, Moujehed et al. (2022) reported that Lip2 lipase is produced from the yeast *Yarrowia lipolytica*. It is effective in degreasing sheepskins, and Lip2 uses only 6 mg/kg of raw skin and is capable of decreasing even in 15 min at pH 8.0 and 30°C successfully.

### 8.4. In pulp and paper production

The pulp and paper industries are growing rapidly around the world and have a crucial role in the economic, social, and environmental development of any country. The increase in population and economic development will also increase the demand for paper. The paper-making process involves raw materials that can be categorized into three groups, namely, wood, non-wood, and recycled waste paper (Abd El-Sayed et al., 2020). In paper industries, some chemical compounds, such as hydrogen peroxidase, sodium carbonate, sodium hydroxide, sodium silicate, diethylenetriaminepenta-acetic acid, and surfactants, are used in high amounts in different steps of conventional methods; these chemicals are very toxic and become highly pollutant in the environment after releasing in wastewater (Yakubu et al., 2019). Several enzymes, such as lipase, amylase, esterase, cellulase, xylanase, hemicellulase, and pectinase, are used as a substitute for these chemicals to reduce toxic waste. Wood is the brassy reservoir of paper pulp and pitch that contains hydrophobic matter, such as triglycerides and waxes, and the presence of this matter creates a very difficult problem in the processing of paper pulp. These triglycerides and waxes might be present as holes or spots and sticky deposits in the finished product (Yakubu et al., 2019).

Therefore, the use of lipases can remove the pitch from the pulpy matter during the paper manufacturing process, and ~90% of present glycerides in the pitch get dissolved into diglycerides, monoglycerides, free fatty acids, and glycerol by lipases, which are less sticky and more hydrophilic (Jaeger and Reetz, 1998). Nippon paper industries in Japan introduced a method to sort out the pitch problem of wood by using the *Candida rugosa* lipase, which can reduce about 90% of triglyceride from the pitch. In Jujo's paper company, Hata and Coworkers in1990 noticed that lipases could reduce pitch problems by reducing the content of triglycerides in ground wood pulp. In another study, *Candida cylindracea* produced lipase that when added to the groundwood stock chest could reduce pitch and talc consumption substantially (de María et al., 2005; Mehta et al., 2017).

### 8.5. In the textile industry

Textile industries are the oldest industries that have existed for many centuries, and they contribute considerably to the economy of several developing countries. Textile industries also help to fulfill one of the introductory demands of humankind (Rahman et al., 2020; Kumar et al., 2021). These industries are facing remarkable environmental and resource challenges, because they are using an intensive amount of chemicals and are thus the most polluting industries. Some chemicals are carcinogenic and have an allergic effect that affects human health. These industries produce waste with chemicals, such as formaldehyde, chlorine, dyes, detergents, and heavy metals (lead and mercury), which cause complex environmental problems (Hooda, 2020; Kumar et al., 2021). To replace these chemicals and overcome these environmental problems, textile industries are now shifting from conventional methods to biological methods that involve the application of enzymes, as they are reliable, stable, safe, and biodegradable; thus, they are widely used in place of toxic chemicals, creating an eco-friendly environment, and they also help in improving the quality of manufacturing products (Kumar et al., 2021). The use of fungal lipases has become crucial in textile industries, and fungal lipases are also used to help in the removal of size lubricants to provide the fabric with better absorbency for raised levelness in dyeing. Commercial preparations also contain lipase enzymes that are used for the desizing of denim and other cotton fabric (Hasan et al., 2006; Mehta et al., 2017). In the textile industry, polyester is a synthetic fiber with certain distinct advantages, such as softness, high strength, strain, wash ability, stretch machine abrasion, and wrinkled resistance; when the polyester fiber is treated enzymatically by lipase, it assists in improving its ability to absorb the dyes and cationic chemicals that support tissue preservation. Lipase produced from *Aspergillus oryzae* is used to modify the polyethylene terephthalate (PET) fabric by increasing its polarity and anti-static ability (Mehta et al., 2017; Kumar and Kumar, 2020). In another study, El Menoufy et al. (2022) reported that the fungus *Aspergillus niger* NRRL-599 produced a lipase through solid-state fermentation and produced an immobilized lipase by using gelatin-coated titanium nanoparticles. The immobilized lipase is used in the treatment of wool after dyeing to improve color and strength.

### 8.6. In biosensor development

Quantitative determination of triglycerides, mainly fats and oils, in the food industry and clinical diagnosis are of high importance, and this function can be performed by the biosensor, which is a lipid sensing device that is cheaper and faster working compared to the chemical methods of determination of triglycerides (Mehta et al., 2017). A biosensor device based on the enzyme-catalyzed dissolution of biodegradable polymer films has been formed (Singh and Mukhopadhyay, 2012). Sumner et al. (2001) have investigated three polymerase-enzyme systems, namely, a dextran hydrogel, degraded by the enzyme dextranase; a poly (ester amide), degraded by the protein degrading α-chymotrypsin; and a poly (tri-methylene) succinate, degraded by a lipase enzyme. The basic concept behind the lipase biosensor is to quantify the glycerol released from triglycerides in an analytical sample by the use of enzymatic, colorimetric, and chemical methods (Hasan et al., 2006; Pérez et al., 2019). Moreover, fungus and yeast lipases along with glucose oxidase immobilized on pH/oxygen electrodes can be used as lipid biosensors and for the estimation of triglycerides and blood cholesterol (Imamura et al., 1989). In addition, fungus lipases as biosensors are also used to estimate the level of lipids in clinical diagnosis. For instance, *Candida rugosa* produced a lipase that has been developed as a DNA probe (Benjamin and Pandey, 2001). The lipase from *Candida rugosa* was immobilized on the aluminosilicate, which was used for the detection of organophosphate insecticides such as diazinon in a liquid medium (Zehani et al., 2014). In another study, the lipase of *Candida rugosa* acts as a biocatalyst for the hydrolysis of triglycerides into glycerol and fatty acid and is used as a biosensor for the detection of esters (β-hydro acid) and triglycerides in blood serum (Califano et al., 2014). Lipase, when immobilized at the Nafion membrane onto a graphite–epoxy transducer, can be used for the quantitative determination of glycerides in food samples (Escamilla-Mejía et al., 2015).

### 8.7. In cosmetics and personal care products production

Lipases have played a significant role in the cosmetic sector and personal care products such as softening, cleaning, aroma, and coloring. This sector has a considerable market value after the food and pharma sectors (Marion and Oliver, 2013; Mehta et al., 2017). Lipases have high potential uses in perfumeries and cosmetics, as they show activities in surfactants and aroma production (Mehta et al., 2017). The esterification of glycerol produces mono- and diglycerol that are used as surfactants in the cosmetics and perfume industries. Lipases catalyze the transesterification of 3,7-dimethyl-4,7-octadien-1-ol, which produces rose oxide, and it is a very important fragrance ingredient in the perfume industry (Izumi et al., 1997). Moreover, Mouad et al. (2016) have reported that immobilized *Rhizomucor miehei* lipase is used as a biocatalyst in making personal care products such as skin creams and bath oils. In addition, *Candida antarctica* B yields a lipase, which synthesizes amphiphilic compounds that attain great attention in the cosmetic industry because they have a range of beneficial characteristics for the skin (Mouad et al., 2016).

Unichem International (Spain) has produced isopropyl palmitate, isopropyl myristate, and 2-ethylhexyl palmitate for use as an emollient in personal care products such as skin and sun tan creams and bath oils, and the company also claims that the use of lipases as a substitute for conventional acid as a catalyst can improve the quality of the product with minimum downstream refining (Hasan et al., 2006). Lipases are used in the production of hair waving and also as an ingredient of topical anti-obese creams or in oral administration (Mehta et al., 2017). Vitamin A (retinol) and its derivatives have been used in large amounts in pharmaceuticals and cosmetics, such as skin care products, as water-soluble derivatives of retinol are prepared with the catalytic reaction of the immobilized lipase (Maugard et al., 2002; Mahfoudhi et al., 2022). Nippon Oil and Fats Co. Ltd. was granted a patent for the formulation of propylene glycerol mono-fatty acid ester in the presence of lipase enzyme; this ester is used as an emulsifier and pearling agent in the cosmetic and food industries (Kim et al., 1997). In other research, Khan and Rathod (2020) synthesized n-butyl palmitate (a significant valuable ingredient of cosmetics) from *Candida antarctica* B lipase after covalent immobilization on hydrophobic and macroporous polyacrylate beads in a solvent-free medium.

### 8.8. In medical and pharmaceutical applications

The application of lipases as diagnostic tools is growing rapidly, as their increased levels show the indication of several diseases and also act as important drug targets or marker enzymes in the medical field. The levels of lipase in the blood act as a diagnostic tool for the detection of some pathological concerns such as pancreatic injury and acute pancreatitis (Mahfoudhi et al., 2022). Lipase is the preliminary enzyme for fat metabolism, and its deficiency causes dangerous health problems. Lipase acts as an activator of tumor necrosis factor and assists in the treatment of malignant tumors, and the determination of the lipase level is also significant in the diagnosis of heart ailments (Singh and Mukhopadhyay, 2012). Moreover, lipases isolated from bacteria, fungi, yeast, and some protozoa have been used in the medical and pharmaceutical fields. *Candida rugosa* produces a lipase that is immobilized on nylon supports in the presence of organic solvents; the immobilized lipase is used in synthesizing lovastatin, a widely used drug in the treatment of serum cholesterol reduction (Yang et al., 1997; Mahfoudhi et al., 2022). Similarly, Sharma and Kanwar (2014) reported that the fungus *Serratia marcescens* produces a lipase that can yield a chiral 3-phenyl glycosidic acid by enantioselective hydrolysis, and this intermediate compound is used in the synthesis of diltiazem hydrochloride, a drug used as a coronary vasodilator in many countries (Pérez et al., 2019). Similarly, lipase can emulsify fats together with proteases and could be used in the treatment of digestive tract diseases (Hasan et al., 2006). In another study, it has been noticed that lipases isolated from yeast and fungi can be used as a therapeutic agent for the treatment of gastrointestinal disturbances, dyspepsia, and cutaneous manifestations of digestive allergies (Mehta et al., 2017).

### 8.9. In the synthesis of biodegradable biopolymer and esters

Biopolymers, such as polysaccharides, polyphenols, and polyesters, indicate a great degree of diversity and complexity, and these polymers have attained the focus of many researchers, as they are synthesized from natural renewable resources and are also biodegradable (Pérez et al., 2019). Interestingly, fungal lipases are exploited as biocatalysts in the production of biodegradable compounds. The esterification of oleic acid and butanol results in the synthesis of 1-butyl oleate, which reduces the viscosity of biodiesel during its use in the winter (Hasan et al., 2006). Similarly, a mixture of 2-ethyl-1-hexyl esters is achieved in an appropriate amount by enzymatic transesterification of rapeseed oil fatty acids and functions as a solvent (Singh and Mukhopadhyay, 2012).

In addition, lipases not only act as biocatalysts in biodegradable biopolymer synthesis but also in ester synthesis in transesterification reactions in organic solvents. The esters of short-chain fatty acids are used as flavoring agents in the food industry (Mehta et al., 2017). The *Candida rugosa* lipase catalyzes the esterification reaction between fatty acids with sulcatol in toluene (Janssen et al., 1999). In a study, after physical adsorption of the lipases of *Mucor javanicus* (MJL), *Candida rugosa* (CRL), and *Candida* sp. (CALA) on a Diaion HP-20 (hydrophobic and mesoporous support), these lipases were used as biocatalysts in the synthesis of the aromatic esters geranyl butyrate, hexyl butyrate, and propyl butyrate, respectively (Dos Santos et al., 2021). The esterification reaction between lauryl alcohol and palmitic acid has also used the lipase from *Candida antarctica* (Novozym 435) as a catalyst, and thus it is capable of producing more than 90% of lauryl palmitate under optimized conditions (Syamsul et al., 2010). Moreover, the lipase isolated from the *Aspergillus ibericus* has applications as a catalyst in esterification reactions and aroma ester production (Oliveira et al., 2016).

In another study, Mehta et al. (2020) reported that the esterification of ethanol and acetic acid produces ethyl acetate, while the esterification of ethanol and lactic acid gives ethyl lactate; both reactions are catalyzed by the lipase of *Aspergillus fumigatus*.

### 8.10. In ester biodegradation

The application of lipases in the biodegradation of esters is a new developing approach in biotechnology. Lipases can biodegrade aliphatic and aromatic esters through their catalytic actions. Lipases also act as catalysts for transesterification reactions in an organic solvent, through which they catalyze the biodegradation of polyesters (Pérez et al., 2019). The lipase-mediated degradation of a polymer is a robust substitute approach to conventional methods because of its biocompatibility and mild conditions. Lipase produced from *Penicillium fellutanum* can degrade polyester Nylon-200 by ~80% by weight in 7 days (Amin et al., 2021). Another study was carried out by da Costa et al. (2020) in which they observed that *Yarrowia lipolytica* produces a lipase that can degrade aromatic ester polyethylene terephthalate (PET).

### 8.11. In the bioremediation of wastewater

The lipase-mediated bioremediation of wastewater is a novel, robust, and extensively used waste management technique in lipase biotechnology. Lipases have applications in activated sludge and other aerobic waste processes where thin layers of fats are continuously removed from the surface of aerated tanks to maintain the oxygen supply; this skimmed fast rich liquid is digested by the lipases (Cruz et al., 2020). Fungal sp. can be applied for degrading oil spills in the coastal environment, which may enhance ecorestoration and also help in the enzymatic processing of oil in industries (Gopinath et al., 1998). Species that belong to the genera *Aspergillus, Fusarium, Cladosporium, Trichoderma, Mortierella, Penicillium, Beauveria*, and *Engyodontium* are examples of fungi that have recently been designated as potential bioremediation agents in soil (Islam and Datta, 2015). The lipase of *Candida rugosa* has applications as an anaerobic digester (Singh and Mukhopadhyay, 2012). Lipases are involved in the effective breakdown of solids and the removal and prevention of fat blockages or filming in the waste system in several industrial processes, such as the treatment of sewage, the cleaning of holding tanks, septic tanks, and grease traps, and P (Singh and Mukhopadhyay, 2012). *Aspergillus terreus* and *Aspergillus niger* produce lipases that are used in the bioremediation of polluted soil and the degradation of polyvinyl alcohol films, respectively (Mahmoud et al., 2015; Mehta et al., 2017). Lipases of *Aspergillus uvarum* and *Aspergillus ibericus* also have applications in bioremediation processes (Salgado et al., 2016). Leal et al. (2006) noticed that synthetic mobile dairy wastewater with or without pre-hydrolysis, is treated anaerobically by solid peprations of *Penicillium restrictum* and *Aspergillus niger*, both of which were isolated from oil-polluted soil and tested for lipase production. The obtained lipase was found to biodegrade polyaromatic hydrocarbons present in petroleum-contaminated soil (Mauti et al., 2016). Basheer et al. (2011) isolated a fungus *Aspergillus awamori* BTMFW032 from seawater, which was able to produce lipase and could reduce ~92% of the fat and oil contents from the mill effluent containing waste oil. A lipase isolated from *Geotrichum candidum* has applications in bioremediation and controlling the decolorization of olive mill wastewater (Kumar et al., 2020).

## 9. Application of CRISPR/Cas genome editing technology to modify lipase encoding genes

Genome editing is a form of genetic engineering in which DNA is purposefully added, subtracted, or altered in living cells. The acronym CRISPR (clustered regularly interspaced short palindromic repeat) refers to the special arrangement of brief, partially repeated DNA sequences present in the prokaryotic genomes. Prokaryotes use CRISPR and its associated protein (Cas9) as a form of adaptive immunity to protect themselves against viruses or bacteriophages (Hille and Charpentier, 2016; Asmamaw and Zawdie, 2021).

The three fundamental steps of the CRISPR defensive system, namely, adaptation (spacer acquisition), crRNA synthesis (expression), and target interference, protect bacteria from recurrent viral infections. CRISPR loci are a collection of brief repetitive sequences that are present in prokaryotic chromosomal or plasmid DNA. The Cas gene, which produces the nuclease protein (Cas protein) necessary to break or destroy viral nucleic acid, is typically found adjacent to the CRISPR gene (Rath et al., 2015).

Nearly, 50% of bacteria, about all the archaea, and even some bacteriophages hold the genes essential for CRISPR-Cas adaptive immunity, which supplies a memory of previous infections through encoding short sequences of DNA at clustered regularly interspaced short palindromic repeat (CRISPR) loci within their genome. These previous infections are stored as spacer sequences, each of which is flanked by repeat sequences. Firstly, these spacer sequence repeats are transcribed into pre-crRNA and then processed into functional crRNAs (Jiang and Doudna, 2015; McCarty et al., 2020). Inherently, the native CRISPR-Cas system is multiplexed; an organism can encode one or several CRISPR arrays and also express many Cas (CRISPR-associated) proteins that help the acquisition of new spacers and further processing of the CRISPR arrays (McCarty et al., 2020). By using Cas proteins and a library of CRISPR RNAs (crRNAs) from CRISPR loci, the CRISPR-Cas system can detect and silence foreign nucleic acids through sequencing and RNA editing. The invasive (non-self) DNA is targeted by CRISPR-Cas via base pairing with the crRNA guide sequence, leading to Cas protein-mediated DNA cleavage (Barrangou et al., 2007; Garneau et al., 2010).

Fungal research has recently been revolutionized by CRISPR/Cas9 genome-editing technology. The first CRISPR/Cas9 genome editing system was introduced to *Saccharomyces cerevisiae* by DiCarlo et al. (2013). Consequently, Liu et al. (2015) applied the CRISPR/Cas9 genome-editing system to *Trichoderma reesei*; Matsu-Ura et al. (2015) and Nodvig et al. (2015) applied this system to the model fungi *N. crassa* and *A. nidulans*, respectively. Since then, the CRISPR/Cas9 genome-editing system has found widespread applications in the genetic alteration of many filamentous fungi, including numerous vital genera such as *Neurospora, Trichoderma, Penicillium*, and *Aspergillus* (Jin et al., 2022). This genome-editing strategy and its application have been well-established, particularly in *Aspergilli*.

Liu et al. (2017) successfully engineered the genome (gene level) of *Myceliophthora thermophila* and Myceliophthora heterothallic with the CRISPR/Cas9 technique and found that engineered fungal sp. is able to produce significantly increased (5- to 13-fold) cellulase. Similarly, in this way, we can engineer the genomes (by adding high-potential lipase genes) of lipase-producing fungi with the CRISPR/Cas9 system to increase the lipase production ability of these fungi. Li et al. (2023) modified the genome of *Aspergillus oryzae* with CRISPR/Cas9 technology, and the heterologous lipase gene (TLL) achieved the precise integration of different genetic loci in one step.

## 10. Conclusion and future prospects

Lipases have been found and characterized in a wide range of fungi and yeasts and act as biocatalysts in the hydrolysis of oils and fats (triglycerides) into fatty acids and glycerol. Lipases are also recognized as having applications in different industries and sectors such as bioethanol production, pulp and paper production, biosensor development, the production of cosmetics and personal care products, food processing, the textile industry, the medical and pharmaceutical sector, bioremediation of wastewater, biodegradable biopolymer synthesis, ester synthesis, and ester biodegradation. Lipase immobilization could improve the thermal or ionic stability, easy recycling, and volume-specific loading of enzymes onto the support, all of which lead to better activity of lipases. Advanced techniques such as genetic engineering also enhance the increased production of lipases in fungi.

Therefore, encouraging the isolation of new fungal species with a high yield of lipase is needed in order to meet the industrial and domestic requirements of lipases in the near future. New optimization techniques to induce high levels of fungal lipase production with better quality must also be sought after. To improve the immobilization techniques for better performance, lipases as diagnostic tools in the medical sector should be further investigated. The application of fungal lipases in bioremediation should also be studied more and can be carried out on a large scale. Moreover, advanced techniques, such as genetic engineering and molecular techniques, should be used profusely to improve production and quality. Genome editing in fungal strains through the CRISPR/Cas9 technique may be used for improving the production and application of lipases in various fields.

## Author contributions

All authors listed have made a substantial, direct, and intellectual contribution to the work and approved it for publication.
